# Health anxiety during a global pandemic: a comparison of medical and non-medical students in Mashhad, Iran

**DOI:** 10.3389/fpsyt.2024.1466026

**Published:** 2024-11-28

**Authors:** Mahsa Nahidi, Mohammad Reza Fayyazi Bordbar, Hanieh Mohammadi, Negar Morovatdar, Maryam Emadzadeh, Hassan Mirshafiei

**Affiliations:** ^1^ Psychiatry and Behavioral Sciences Research Center, Mashhad University of Medical Sciences, Mashhad, Iran; ^2^ Faculty of Medicine, Mashhad University of Medical Sciences, Mashhad, Iran; ^3^ Clinical Research Development Unit, Faculty of Medicine, Mashhad University of Medical Sciences, Mashhad, Iran; ^4^ Clinical Research Development Unit, Ghaem Hospital, Mashhad University of Medical Sciences, Mashhad, Iran

**Keywords:** COVID-19, pandemic, health anxiety, medical students, mental health

## Abstract

**Background:**

The COVID-19 pandemic has negatively impacted mental health worldwide, especially among healthcare professionals, including medical students, who were more exposed to pandemic-related stressors. However, health anxiety within this vulnerable group remains understudied.

**Objective:**

This study aimed to assess and compare health anxiety and COVID-19 anxiety between medical and non-medical students during the COVID-19 pandemic and to identify factors associated with these forms of anxiety.

**Methods:**

This cross-sectional study recruited Iranian medical and non-medical students studying in Mashhad via convenience sampling using messaging apps. Participants completed a self-reported questionnaire on demographic and social factors, along with the Health Anxiety Inventory (HAI) and the Corona Disease Anxiety Scale (CDAS), with higher scores reflecting greater symptom severity. Statistical analyses evaluated group differences, correlations between HAI and CDAS scores, and the influence of confounding variables.

**Results:**

A total of 305 students participated, with 176 medical students (57.7%) and 129 non-medical students (42.3%). The majority (92.7%) reported mild COVID-19 anxiety, while 3.2% reported moderate and 0.98% reported severe COVID-19 anxiety. COVID-19 anxiety did not significantly differ between medical and non-medical students (P = 0.439). However, medical students reported significantly higher fear of illness consequences than non-medical students (P = 0.037), while no significant differences were found in susceptibility to disease (P = 0.299) or general health concern (P = 0.156). HAI and CDAS scores were significantly correlated (r = 0.30, P < 0.001). Based on logistic regression, Female gender (OR = 4.55, P = 0.002) was associated with susceptibility to health anxiety, while studying a non-medical major was associated with lower health anxiety (OR = 0.01, P < 0.001) and lower COVID-19 anxiety (OR = 0.05, P < 0.001).

**Conclusion:**

Mild COVID-19 anxiety was prevalent among both medical and non-medical students, with comparable levels of health anxiety across the groups. These findings suggest the need for targeted mental health support among students during pandemic conditions.

## 1 Introduction

Health anxiety (HA) is defined as a preoccupation with the thought that one has, or is developing, a serious illness despite medical evidence to the contrary (1). People with health anxiety (HA) may misinterpret bodily sensations and changes as signs of illness, potentially leading to a number of cognitive, affective, behavioral, and perceptual impairments such as difficulty concentrating, irritability, anxiety, and difficulty sleeping (2). HA resembles the somatic symptom disorder and illness anxiety disorder, but these conditions are more severe, characterized by excessive and impairing preoccupation with having or acquiring a serious illness, persistently high levels of anxiety about health, and excessive health-related behaviors such as repeatedly seeking medical care despite reassurance from healthcare providers (3, 4). On the other hand, people with HA do not believe they suffer from a serious illness but worry about developing one. They may consult with healthcare professionals repeatedly to seek reassurance, but are usually able to acknowledge that their worries may be unfounded (5). Hypochondriasis is rare but HA affects 2.1-13.1% of the general population (6). HA is equally common at all ages, but it is more prevalent among adults in their 20s and 30s and women (7). HA co-occurs with other mental health disorders and is a prognostic factor for other psychiatric conditions (8). Changes in health perspectives and unhealthy internet use patterns may be responsible for the recent increase in HA (9). HA can lead to serious disability and excess medical service utilization, which can put a strain on medical services. Identifying people with HA and providing them with appropriate treatments can help reduce the burden on healthcare services and personnel (10).

Healthcare professionals are more likely to report mental illness, including HA, due to factors such as increased exposure to patients’ pain, illness, and death, long and irregular working hours, and heavy physical strain. The COVID-19 pandemic amplified the stressors faced by healthcare workers, along with presenting new stressors such as the risk of death and disability due to COVID-19 (11). This has led to an increase in complaints of stress, depression, and anxiety among healthcare workers, making HA more common in this group (12).

A number of studies have explored HA in healthcare professionals in Iran. Bahmaei et al. (2022) investigated HA among 600 medical students in Iran before and during the COVID-19 pandemic. Initially, 47% of medical students reported severe anxiety, which significantly increased to 84% during the pandemic. The overall HA score significantly rose in the same period (13). Javadi et al. (2022) surveyed 735 healthcare workers and volunteers in Iran during April-May 2020, finding that health anxiety was correlated with social health (14). In a study of 101 healthcare professionals working with COVID-19 patients, Mirzabeigi et al. (2021) found that anxiety levels were mild in 72.3%, moderate in 24.8%, and severe in 3% of the participants. Male respondents and those who had witnessed patient deaths had significantly higher HA scores. HA was also higher among participants with less work experience (15). Another study by Mousavi et al. (2022) examined HA among 340 nurses between April and May 2020, reporting that 91.76% of the participants experienced moderate HA. The level of anxiety was correlated with gender, marital status, parenting, family relationships, work experience, and workplace (16). Hassannia et al. (2020) compared the Iranian general public and healthcare workers with respect to depression and anxiety during the COVID-19 pandemic, finding significantly higher prevalence among the healthcare workers (17). Heidari et al. (2020) found that 28.82% of citizens of Mashhad, Iran, experienced corona disease anxiety in the spring of 2020, with significantly higher levels among women and those with lower educational attainment (18). Finally, Mohamadzadeh Tabrizi et al. (2022) studied corona disease anxiety among 1131 Iranian nurses from April to May 2020. The results revealed that 33.4% of the participants experienced moderate anxiety and 13.4% experienced severe anxiety; anxiety was also negatively correlated with quality of life (19).

Still, more research is needed on HA among healthcare workers, especially during the COVID-19 pandemic. Researchers should prioritize this issue to better understand and address the needs of this vulnerable population (20). During their clinical training, medical students experience the same stressors as medical staff and often experience a phenomenon known as “medical student syndrome,” a form of HA characterized by the fear of developing diseases they study and exhibiting related symptoms. A recent study revealed that approximately 11% of medical students encounter this condition during their training. However, previous research has reported a significantly higher prevalence, exceeding 70%. This discrepancy highlights the need for further investigation into the factors contributing to HA among medical students and the development of effective interventions to address this issue (21). To this aim, this study was conducted to compare HA in medical and non-medical students and uncover the correlated factors during the COVID-19 pandemic.

## 2 Method

### 2.1 Setting and approval

This cross-sectional study was conducted during a 12-month period in the 2022-2023 academic year in Mashhad, Iran. After approval by the ethics committee of Mashhad University of Medical Sciences (MUMS) (registration code: IR.MUMS.MEDICAL.REC.1400.317), participants were recruited through convenience sampling and questionnaires were distributed online using messaging apps (i.e., WhatsApp and Telegram).

The inclusion criteria were: being at least 18 years old, being a medical student (for the medical group) or an undergraduate or graduate student (for the non-medical group, including those studying engineering, humanities, law, etc.), and access to WhatsApp or Telegram. To minimize the effect of the stress associated with major exams, medical students had to have completed the questionnaires at least a month before and after major exams such as the Comprehensive Basic Medical Sciences Exam. The exclusion criteria were: failure to complete all questionnaires, and a history of serious mental health conditions.

### 2.2 Data collection

After obtaining informed consent and providing assurance regarding the anonymity of the responses, demographic and social information, including age, gender, marital status, number of children, place of residence, and level of education were collected using a questionnaire. Personal and family history of psychiatric disorders, psychiatric medication use, substance use, history of COVID-19 infection for the respondent or first-degree family members, death of family members and relatives due to COVID-19, and COVID-19 vaccination status were also collected. Finally, the participants completed the Farsi versions of the health anxiety inventory (HAI) and the corona disease anxiety scale (CDAS).

#### 2.2.1 Health anxiety inventory

Health anxiety inventory (HAI) was introduced in 1989 in its long form to develop a cognitive model of health anxiety and hypochondriasis. The short form of HAI was developed by Salkovskis and Warwick in 2002, consisting of 18 questions that assess susceptibility to disease, consequences of disease, and general health concern. HAI evaluates HA using multiple choice questions, with each option assigned a score of 0-3. Participants are asked to choose the answer that most closely matches their experience. Scores <26, 26-34, and >34 are categorized as mild, moderate, and severe health anxiety, respectively. The reliability of HAI for the Iranian population has been confirmed in a study involving 1395 participants (Cronbach’s α = 0.75) (22).

#### 2.2.2 Corona disease anxiety scale

Corona disease anxiety scale (CDAS) was developed and validated in Iran following the COVID-19 pandemic to assess the level of anxiety associated with corona virus infection. The scale includes items assessing psychological (items 1-9) and physical (items 10-18) symptoms. The responses are evaluated on a 4-point Likert scale (never = 0, sometimes = 1, often = 2, and always = 3). Higher scores on this questionnaire indicate higher levels of anxiety. The reliability and validity of this instrument have been confirmed previously, with Cronbach’s α > 0.85 for the two sub-sections and the entire questionnaire (23).

### 2.3 Statistical analysis

Given the lack of similar studies comparing the anxiety levels between these groups, we used the data from the study by Kibbey et al. (24) in which about 15% of nursing student had high anxiety levels during COVID-19 pandemic. Following the researches’ assumption in which the anxiety rate would be about 15% lower in non-medical students, and keeping an α of 0.05 and a β of 0.2, the sample size was calculated as 121 students in each group (medical and non-medical) using the PASS software (NCSS LLC, Kaysville, UT). Aiming to reach that number while considering a potential response rate of 60%, we distributed the questionnaires among 200 students in each group (medical and non-medical) via online messaging apps.

Data analysis was performed in SPSS 26 (IBM Statistics, Chicago, IL). Qualitative variables are presented using frequency and percentage and compared using the Chi-square test or Fisher’s exact test. Quantitative variables are presented using mean and standard deviation (SD) and compared using the independent samples t-test or the Mann-Whitney test. Spearman’s correlation coefficient was used to evaluate the correlation between total HAI and CDAS scores. Furthermore, a logistic regression model was utilized to determine the influence of confounding variables on HAI and CDAS scores. All P-values are reported for two-tailed tests and P < 0.05 was considered statistically significant.

## 3 Results

A total of 305 individuals completed the survey during the 12-month data collection period (response rate: 76.25%). The non-medical students group consisted of 129 participants with a mean age of 26.05 ± 9.25 years, while the medical students group consisted of 176 participants with a mean age of 24.44 ± 2.44 years. In terms of gender distribution, the medical students had a higher percentage of females (73.3%) compared to the non-medical students (58.1%), with females being significantly overrepresented in the medical groups (P = 0.005). There were no statistically significant differences between the two groups with regards to marital status, place of residence, history of substance use, and personal and family history of anxiety disorders (**Table 1**).

**Table 1:** Demographic characteristics and medical history of the participants.

Variable	Medical Students (N=176)	Non-Medical Students (N=129)	P
**Age (years)**	24.44 ± 2.44	26.05 ± 9.25	0.056*
**Gender (female)**	129 (73.3%)	75 (58.1%)	0.005**
Marital status			
Single	128 (72.7%)	96 (74.4%)	0.741**
Married	48 (27.3%)	33 (25.6%)	
Place of residence			
Dormitory	46 (26.1%)	26 (20.1%)	0.568**
With parents	70 (39.7%)	60 (46.5%)	
Personal housing	58 (32.9%)	42 (32.5%)	
Other	2 (1.1%)	1 (0.7%)	
**Substance use**	14 (8%)	13 (10.1%)	0.519**
**Personal history of anxiety disorders**	43 (24.4%)	34 (26.3%)	0.702**
**Family history of anxiety disorders**	57 (32.3%)	51 (39.5%)	0.197**
**Personal history of COVID-19**	151 (85.7%)	96 (74.4%)	0.012**
**Family history of COVID-19**	163 (92.6%)	111 (86%)	0.060**
**COVID-19 mortality in family and relatives**	75 (42.6%)	75 (58.1%)	0.007**
**Vaccination status (vaccinated)**	176 (100%)	122 (94.5%)	0.002***

Values are reported as mean ± standard deviation (for quantitative variables) or frequency and percentage (for categorical variables).

*Independent samples T-test.

**Chi-square test.

***Fisher’s exact test.

The bold values represent statistical significance.

Regarding COVID-19 variables, a higher percentage of medical students (86.4%) reported a personal history of COVID-19 infection compared to non-medical students (74.4%), with this difference being statistically significant (P = 0.012). While the difference in family history of COVID-19 was not statistically significant (P = 0.060), there was a significant difference in the percentage of participants who reported COVID-19 mortality in their family or among relatives, with 42.6% of medical students being affected compared to 58.1% of non-medical students (P = 0.007). Additionally, all medical students were vaccinated against COVID-19, whereas a small percentage of non-medical students (5.4%) were unvaccinated, representing a statistically significant difference (P = 0.002). **Table 1** presents the demographic characteristics and medical history of the participants.

### 3.1 Health anxiety

**Table 2** presents the data on HA among the participants. The “Susceptibility to disease” subscale did not show a significant difference between the two groups, with mean scores of 3.90 ± 3.27 for non-medical students and 4.05 ± 2.66 for medical students (P = 0.299). However, there was a statistically significant difference (P = 0.037) in the “Consequences of disease” subscale; medical students had a higher mean score (5.01 ± 2.70) compared to non-medical students (4.54 ± 3.28). The “General health concern” subscale did not show a significant difference between the two groups (P = 0.156), with mean scores of 5.60 ± 2.69 for medical students and 5.17 ± 3.16 for non-medical students (**Table 2**).

**Table 2:** Analysis of responses to HAI, compared between medical and non-medical students.

Variable	Medical (n=176)	Non-medical (n=129)	P*
**Susceptibility to disease**	4.05 ± 2.66	3.90 ± 3.27	0.299
**Consequences of disease**	5.01 ± 2.70	4.54 ± 3.28	0.037
**General health concern**	5.60 ± 2.69	5.17 ± 3.16	0.156
**Total score**	17.36 ± 2.65	15.25 ± 3.25	0.244

*Mann-Whitney test.

The bold values represent statistical significance.

### 3.2 Corona disease anxiety

**Table 3** presents the data on corona disease anxiety levels among the participants. The majority of both groups reported mild levels of anxiety, with 56.8% of medical students and 58.1% of non-medical students falling into this category. Similarly, similar numbers reported moderate psychological symptoms in the two groups. Only a small proportion reported severe psychological symptoms (1.1% for medical students and 1.6% for non-medical students), with no statistically significant difference between the groups (P = 0.917). In terms of physical symptoms, most participants in both groups experienced mild levels, with 85.2% of medical students and 75.2% of non-medical students falling into this category. The percentage of participants with moderate physical symptoms was higher among non-medical students (23.3%) compared to medical students (13.6%). However, the proportion of individuals reporting severe physical symptoms was low and identical (0.7%) for both groups, with no statistically significant difference between the groups (P = 0.088). Regarding overall COVID-19 anxiety levels measured by the total score, most students in both groups exhibited mild anxiety. Specifically, 92.0% of medical students and 93.8% of non-medical students fell into the mild category for the total score. A moderate level of total anxiety was more prevalent among medical students (7.4%) compared to their non-medical counterparts (4.7%). Severe total anxiety scores were relatively uncommon, with only 0.6% of medical students and 1.6% of non-medical students falling into this category. However, the difference in the distribution of total anxiety scores was not statistically significant (P = 0.439) (**Table 3**).

**Table 3:** Analysis of responses to CDAS.

Corona disease anxiety		Medical (n=176)	Non-medical (n=129)	P*
**Psychological symptoms**	Mild	100 (56.8%)	75 (58.1%)	0.917
	Moderate	74 (42.0%)	52 (40.3%)	
	Severe	2 (1.1%)	2 (1.6%)	
**Physical symptoms**	Mild	150 (85.2%)	97 (75.2%)	0.088
	Moderate	24 (13.6%)	30 (23.3%)	
	Severe	2 (0.7%)	2 (0.7%)	
**Total score**	Mild	162 (92.0%)	121 (93.8%)	0.439
	Moderate	13 (7.4%)	6 (4.7%)	
	Severe	1 (0.6%)	2 (1.6%)	

*Chi-square test.

Values are reported as frequency and percentage.

The bold values represent statistical significance.

### 3.3 Correlation between HAI and CDAS and confounding factors

Correlation analysis revealed a moderate but significant positive correlation between total CDAS and HAI scores (P<0.001, r=0.30). **Table 4** presents the results of logistic regression analysis to investigate the effects of gender (female) and group (non-medical student) as confounding variables on total HAI and CDAS scores. Being female was significantly associated with higher odds ratio (OR) for HAI (OR = 4.55, 95% CI = 1.73-11.90, P = 0.002), indicating that female respondents faced approximately 4.5 times greater risk compared to males. Being a non-medical student was significantly correlated with lower HAI scores (OR = 0.01, 95% CI = 0.29-1.90, P < 0.001), suggesting that non-medical students face comparatively lower HA. Regarding the effect of confounding variables on CDAS, the results indicate that gender was not significantly associated with CDAS (P = 0.695 and P = 0.534, respectively). In contrast, being a non-medical student was significantly associated with lower CDAS scores (OR = 0.05, 95% CI = 0.44-2.84, P < 0.001), indicating lower odds of corona disease anxiety for non-medical students (**Table 4**).

**Table 4:** Logistic regression analysis for the effect of confounding variables.

	Confounding variable	β	Odds ratio (OR)	95% CI		P-value*
				Lower	Upper	
**HAI**	Gender (female)	1.50	4.55	1.73	11.90	**0.002**
	Group (non-medical)	-0.28	0.01	0.29	1.90	**<0.001**
**CDAS**	Gender (female)	-0.19	0.81	0.30	2.22	0.695
	Group (non-medical)	-2.94	0.05	0.44	2.84	**<0.001**

*Logistic regression.

The bold values represent statistical significance.

## 4 Discussion

This study evaluated HA among medical and non-medical students during the COVID-19 pandemic. Although the two groups were similar in many regards, the significantly higher percentage of medical students (86.4%) reporting a personal history of COVID-19 infection compared to non-medical students (74.4%) could be attributed to the increased exposure of medical students to healthcare settings and patients during their clinical training. This observation aligns with previous studies that highlight the elevated risk of COVID-19 infection among healthcare workers in Malaysia and Iran (25–27). Interestingly, while the difference in family history of COVID-19 between the two groups was not statistically significant, a higher proportion of non-medical students (58.1%) reported COVID-19 mortality in their family or among relatives compared to medical students (42.6%). This finding could potentially be explained by the higher likelihood of medical students being more informed about COVID-19 prevention measures and having better access to healthcare resources, which may have reduced the risk of severe outcomes or mortality among their family members and relatives.

Vaccination status data revealed that all medical students were vaccinated against COVID-19, while a small percentage (5.4%) of non-medical students remained unvaccinated. This discrepancy could be attributed to the mandatory vaccination policies implemented in many medical institutions and healthcare settings, as well as the heightened awareness and emphasis on vaccination among medical students due to their educational background and contact with healthcare professionals. Moreover, the vaccine roll-out in Iran prioritized healthcare workers due to their exposure to COVID-19 risk.

The exact etiology of health anxiety disorder is unknown, but several factors have been shown to contribute to this disorder, including anxiety sensitivity, body vigilance, intolerance of uncertainty, obsessive-compulsive symptoms, safety behaviors (e.g., frequent doctor visits, online disease research, reassurance-seeking), and a history of medical and mental health conditions (28–30). The findings from HAI provide valuable insights into the differences in health anxiety levels between medical and non-medical students. Notably, the “susceptibility to disease” subscale did not reveal a significant difference between the two groups. However, a significant difference was observed in the “consequences of disease” subscale, with medical students scoring higher than non-medical students. This finding indicates that medical students perceived the consequences of disease as more severe compared to their non-medical counterparts. This could be due to increased exposure to clinical settings and patient cases, greater medical knowledge, and the psychological impact of medical education and work in healthcare settings. Research indicates that working in the healthcare sectors can induce a higher level of anxiety and depression. Medical students are exposed to both disease knowledge and patients suffering from illnesses. Additionally, a systematic review and a cross-sectional study in Poland showed that medical professionals were more likely to report mental health issues such as depression and anxiety, especially during the COVID-19 pandemic (31, 32). Studies have shown that somatic symptoms and anxiety were higher among healthcare professionals during the COVID-19 pandemic, with the highest prevalence figures belonging to the Middle-East and Asia (33, 34). Moreover a cross sectional study on 382 Iranian medical and non-medical students found that medical students are at a higher risk of developing HA and hypochondriasis compared to non-medical students (35). A study involving 193 medical students in the United Arab Emirates (UAE) using the short health anxiety inventory (SHAI) questionnaire reported that HA is a prevalent phenomenon among medical students (30). Similarly, a study conducted on 600 medical students in Iran showed the significant effect of the COVID-19 pandemic on HA among medical students (13). Therefore, it is not surprising that medical students had higher scores on some sub-scales of HAI than the general population, represented by the non-medical students in this study.

At the same time, our results showed no significant difference between the groups in total HAI score. This finding suggests that while medical students have more knowledge about disease consequences, this knowledge does not translate into higher overall HA, contradicting the hypothesis that increased disease knowledge leads to increased anxiety. Several studies have been conducted during the COVID-19 pandemic to compare mental health between medical and non-medical students, and their findings suggest that medical students had comparatively lower levels of anxiety in the UAE, China, and the United Kingdom (36–38). The findings of a study involving Croatian medical, non-medical, and law students contradict the notion that medical students are more inclined to seek assistance due to hypochondriacal thoughts (39). Aligned with this result, a study of 214 medical and 821 non-medical students in China using the health anxiety questionnaire (HAQ) showed that HA is significantly lower in medical students (40). A recent study conducted in Mexico using the patient health questionnaire-9 (PHQ-9) and the general anxiety disorder (GAD) scale reports that non-medical students had more anxiety and depressive symptoms than medical students during the COVID-19 pandemic (41).

The contradictory findings regarding HA in medical and non-medical students could stem from variations in study populations, methodologies, assessment tools, and contexts. While medical students’ disease knowledge may heighten perceptions of consequences, it could also provide better coping strategies and mitigate anxiety. Factors like stage of education, curriculum, and availability of mental health resources could influence observed anxiety levels as well. Cultural, regional, and circumstantial variations, such as the COVID-19 pandemic, may also contribute to divergent results. Reconciling these contradictions requires further research exploring the interplay between medical knowledge, clinical exposure, coping mechanisms, and psychological well-being.

We found that the majority of participants from both groups reported mild levels of COVID-19 anxiety, with no statistically significant differences in the distribution of psychological symptoms or overall anxiety scores. This finding suggests that, despite the varying educational backgrounds and exposure to healthcare settings, the pandemic’s psychological impact was relatively similar for medical and non-medical students. Interestingly, the data on physical symptoms revealed that a higher proportion of non-medical students (23.3%) experienced moderate levels of physical symptoms compared to medical students (13.6%). This observation could be linked to the better understanding of COVID-19 symptoms and the potential influence of medical knowledge on the interpretation and management of physical manifestations among medical students. In terms of corona disease anxiety, a study investigating the psychological impact of the outbreak on medical and non-medical students in China found that while medical students considered COVID-19 to be more serious, they scored lower on the impact of event scale-revised (IES-R) and experienced less anxiety and depression compared to non-medical students (36). Similarly a recent work from Pakistan using the corona anxiety scale (CAS) revealed that non-clinical staff scored higher than clinical staff (42). In line with our findings, a recent study by Nadeem et al. (2020) using the CDAS questionnaire demonstrated that Pakistani healthcare workers have mild coronavirus disease anxiety but report lower levels of anxiety due to COVID-19 compared to the general population (43). However, a study conducted on 385 Ecuadorean participants reported high levels of anxiety in both medical and non-medical students, with a higher prevalence figure for medical students (44). Furthermore, a recent study revealed high levels of corona disease-induced anxiety among Portuguese undergraduate students (45). These findings contradict our results, which indicate mild COVID-19 anxiety among students. It is worth noting that our study was conducted about 1.5 years after the outbreak began, in a context where students had gained greater awareness of the disease and most had been vaccinated against COVID-19 at least once. These factors may help reconcile the discrepancies between our results and those of other studies.

We observed a mild but significant positive correlation between total HAI and CDAS scores, suggesting that individuals with higher levels of health anxiety are more likely to experience increased anxiety specifically related to the COVID-19 pandemic. A study of 1638 Iranians revealed a similar correlation between HA and COVID-19 anxiety (28). Another study, conducted in Portugal using the depression, anxiety, and stress scale-21 (DASS-21), and the coronavirus anxiety scale (CAS), also found a statistically significant correlation between general anxiety and COVID anxiety (46). In the present research, female respondents faced approximately 4.5 times greater odds of HA, which is consistent with previous research indicating a higher prevalence of anxiety disorders among women in general, and during the COVID-19 pandemic specifically, possibly due to the potential influence of biological and social factors (47–49). Being a non-medical student was significantly associated with lower odds of both health anxiety and corona disease anxiety. This finding aligns with the earlier discussions, highlighting the potential influence of medical knowledge and exposure to clinical settings on the perception of disease consequences and subsequent anxiety levels.

The literature on the relationship between field of study and HA contains contradictory evidence regarding the prevalence of HA among medical and non-medical students. The reasons for these discrepancies are not fully understood but may be related to the use of different assessment tools for measuring HA, the different demographics of medical and non-medical students in each study, cultural differences, and different baseline anxiety levels. The present study found wide-spread anxiety among medical and non-medical students, with significant differences between the two groups in some sub-scales. The Mann-Whitney test did not find significant differences between medical and non-medical students with respect to HAI, and the Chi-square test did not reveal any differences with respect to CDAS classes. In contrast, the results of logistic regression analysis indicate the protective effect of being a non-medical student. The seemingly contradictory findings can be reconciled by understanding the different analytical focuses of these methods. The Mann-Whitney and Chi-square tests compare general score distributions without adjusting for other factors, while logistic regression assesses the likelihood of anxiety, adjusting for covariates like gender. Together, these analyses indicate that, although health anxiety levels may not differ overall, non-medical students are less likely to experience high health anxiety when confounding factors are considered.

This study suffers from a number of limitations, including its cross-sectional study design, small sample size, and online data collection. A large number of individuals failed to complete the questionnaires, partly due the collection period coinciding with several months of internet access interruption in Iran. Although we provided a concise introduction accompanying the questionnaire, the lack of familiarity with the significance of this research theme might have also contributed to lower response rates among the non-medical group. Additionally, fewer non-medical students were recruited due to the difficulty of contacting non-medical students. Finally, our study was conducted in the city of Mashhad, which restricts the generalizability of the results to other populations.

## 5 Conclusion

The present study highlights the prevalence of health anxiety among medical and non-medical students. Notably, our results indicate that medical and non-medical students experience similar levels of overall health anxiety, but different levels of anxiety regarding the consequences of illness. We also found that being male and studying non-medical majors exert protective effects against health anxiety. Our findings underscore the importance of psychoeducation and mental health awareness in healthcare and educational settings. Furthermore, efforts to reduce stress and burnout, and promote healthy coping mechanisms and stress management strategies are recommended to improve overall student well-being. These findings emphasize the need for targeted interventions and support systems within educational programs to address the unique challenges faced by different student populations and promote a balanced perspective on health and disease, fostering resilience and mental well-being among future professionals. Moreover, further research is needed to elucidate the effect of field of study on health anxiety.

## Data Availability

The raw data supporting the conclusions of this article will be made available by the authors, without undue reservation.
